# Evaluation of a *Crataegus*-Based Multiherb Formula for Dyslipidemia: A Randomized, Double-Blind, Placebo-Controlled Clinical Trial

**DOI:** 10.1155/2014/365742

**Published:** 2014-04-16

**Authors:** Miao Hu, Weiwei Zeng, Brian Tomlinson

**Affiliations:** Department of Medicine & Therapeutics, The Chinese University of Hong Kong, Prince of Wales Hospital, Shatin, Hong Kong

## Abstract

*Background.* We for the first time examined the effects of a multiherb formula containing *Crataegus pinnatifida* (1 g daily), *Alisma orientalis*, *Stigma maydis*, *Ganoderma lucidum*, *Polygonum multiflorum*, and *Morus alba* on plasma lipid and glucose levels in Chinese patients with dyslipidemia. *Methods.* In this randomized, double-blind, placebo-controlled study, 42 patients were randomized at a ratio of 1 : 1 to receive the herbal formula or placebo for 12 weeks and 40 patients completed the study. Lipid profiles, glucose, glycated haemoglobin (HbA1c), and laboratory safety parameters were performed before and after treatment. *Results.* The difference in the changes in low-density lipoprotein cholesterol (LDL-C) levels between placebo and active treatment (−9%) was significantly (*P* < 0.05) better with active treatment. HbA1c levels significantly decreased by −3.9% in the active treatment group, but the change was not significantly different from that with placebo (−1.1%) (*P* = 0.098). There were no apparent adverse effects or changes in laboratory safety parameters with either treatment. *Conclusions.* The multiherb formula had mild beneficial effects on plasma LDL-C after 12-weeks treatment in subjects with dyslipidemia without any noticeable adverse effects.

## 1. Introduction

Herbal medicines have been used for thousands of years in China and other Eastern countries and have regained popularity in Western Countries in the last two decades [1, 2]. There is growing awareness of the place of dietary factors and herbal medicines in the prevention of cardiovascular disease (CVD) and the possibility of their use in treatment of CVD risk factors [3]. Herbal materials have also resulted in the development of many important conventional drugs, including digoxin and the 3-hydroxy-3-methylglutaryl-coenzyme A reductase inhibitors or statins. More recently, some natural products were found to be effective in the treatment of hyperlipidaemia and diabetes in animal and in human studies [4–6]. Among 57 traditional Chinese medicine (TCM) formulas that have been approved by the China Food and Drug Administration (CFDA) to treat hyperlipidaemia in mainland China, hawthorn fruit (*Crataegus pinnatifida* Bge.), also known as Shan Zha, is the most popular TCM prescribed in more than 50% of the formulas, followed by* Polygonum multiflorum* (or Fo-ti root, known as He Shou Wu in China) (38%) and* Alisma orientalis* (Ze Xie) (33%) [6].

**Figure 1:**
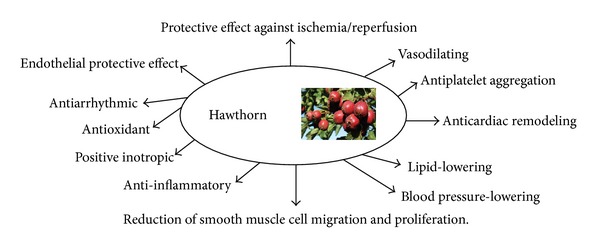
Multiple cardiovascular protective effects of* Crataegus.* Adapted from [9].

*Crataegus *products are widely used for the treatment of cardiac and circulatory disorders, particularly for angina, heart failure, and hyperlipidaemia as they are considered to have multiple cardiovascular protective effects (Figure 1) [7–9].* Crataegus* leaves, flowers, and fruits contain varying amounts of a number of biologically active substances, such as oligomeric procyanidins, flavonoids, and triterpenes [10, 11]. Among these components, flavonoids and triterpenes, especially ursolic acid, have been reported as the main active constituents exerting hypolipidaemic effects [12]. Recent research indicated that the lipid-lowering effect of* Crataegus* may be related to the inhibitory effects of flavonoids on 3-hydroxy-3-methylglutaryl-coenzyme A reductase [10], downregulation of intestinal acyl-coA: cholesterol acyltransferase activity by the triterpenes [13], and activation of peroxisome proliferator activated receptor (PPAR) alpha in adipose tissue [14] or in liver [15]. Furthermore,* Crataegus* extract has been reported to have antioxidant and nitrite reductase activities and inhibits the formation of thromboxane to modify cardiovascular risk factors [16–18]. Although* Crataegus* has been found to decrease the plasma levels of low-density lipoprotein cholesterol (LDL-C), triglycerides, and glucose in various animal models [14, 19–22], limited data are available in humans.

*Polygonum multiflorum* and* Alisma orientalis* are also important components in TCM formulas for the treatment of hyperlipidaemia [6]. Stilbene glycosides extracted from the root of* Polygonum multiflorum* showed antagonistic effects on oxidation of lipoproteins and proliferation of coronary arterial smooth muscle cells and a decrease in their content of nitric oxide [23]. Some animal studies and an early uncontrolled clinical study suggested that* Polygonum multiflorum* has lipid-lowering effects which may be related to its regulatory effects on the genes involved in cholesterol synthesis and lipoprotein metabolism [6].* Alisma orientalis* has been attributed with multiple pharmacological effects, for example, antidiabetic, antihepatitis, and antidiuretic, and it is utilized to treat hyperglycaemia, hyperlipidaemia, and nephritis and for neuroprotection in TCM [24]. It has been reported that triterpenes (alisol M 23-acetate and alisol A 23-acetate) from* Alisma orientalis* act as farnesoid X receptor agonists, which may be responsible for the antidiabetic and other therapeutic effects of* Alisma orientalis* [24]. In rats with experimental nonalcoholic fatty liver disease induced by high-fat diet, administration of* Alisma orientalis* (150, 300, and 600 mg/kg) markedly decreased the serum and liver lipids, decreased plasma glucose levels, improved insulin resistance, and reduced markers of liver injury, aminotransferase abnormalities, and hepatomegaly [25].

Some other herbs such as* Ganoderma lucidum* (Ling Zhi),* Stigma maydis *(corn silk or Yu Mi Xu in Chinese), and* Morus alba L*. (Sang Ye) have also been recommended for a long time for protection of CVD, partly through their potential benefits in reducing plasma lipids and glucose levels [26–28]. In obese diabetic mice, a water extract of* Ganoderma lucidum* lowered the plasma LDL-C levels without affecting the expression of 3-hydroxy-3-methylglutaryl-coenzyme A reductase in both hepatic and extrahepatic organs [29]. Double-blinded, placebo-controlled, and crossover intervention studies in healthy volunteers and in patients with diabetes showed that* Ganoderma lucidum *supplement (1.44 g daily) may improve plasma lipid profiles [30, 31].* Stigma maydis* is rich in phenolic compounds, particularly flavonoids [28]. A recent animal study showed that the flavonoids from* Stigma maydis* extract significantly lowered plasma levels of LDL-C and triglycerides in rats fed high-cholesterol diet indicating that flavonoids from* Stigma maydis* extract may have potential antihyperlipidemic effects [32].* Morus alba L*. has been used in traditional Chinese medicine for cardiovascular, liver, and spleen disorders. Several animal studies in rats have demonstrated that* Morus alba L*. reduced plasma triglycerides, free fatty acid, and/or LDL-C [33, 34]. DNA microarray analysis revealed that mulberry leaves upregulated expression of the genes involved in the peroxisome proliferator-activated receptor signaling pathway and downregulated the genes involved in lipogenesis [33]. In another study in hamsters fed with high fat/cholesterol diets,* Morus alba L*. extract (1% and 2%) significantly reduced plasma total cholesterol and triglyceride levels by 30–37% and 16–35%, respectively [35]. Low-density lipoprotein receptor gene expression and the uptake ability of LDL in HepG2 cells were upregulated, whereas the gene expressions of enzymes involved in triglyceride and cholesterol biosyntheses were decreased with* Morus alba L.* [35]. These studies suggested that* Stigma maydis* and* Morus alba L*. can be used as natural agents against hyperlipidaemia. However, to the best of our knowledge, there is no study reporting the effect of these natural products on plasma lipid profiles in human.

The present study for the first time examined the effect of a commercially available multiherb formula containing the herbs mentioned above (Table 1) on reducing plasma lipid and glucose levels in Chinese patients with dyslipidemia.

## 2. Materials and Methods

### 2.1. Participants

#### 2.1.1. Inclusion Criteria

Subjects aged ≥18 years with dyslipidemia (familial or nonfamilial) were recruited from the patients who were regularly attending the Lipid Clinic in the Prince of Wales Hospital, Hong Kong. Dyslipidemia was defined as either having a documented history of dyslipidemia and receiving lipid-lowering therapy or having a documented elevated baseline fasting LDL-C cholesterol (≥4.1 mmol/L) or triglycerides ≥1.7 mmol/L based on local laboratory reference values. Patients were eligible if they had a plasma level of LDL-C ≥2.6 mmol/L or ≥1.8 mmol/L for those with high cardiovascular risk (because of a history of coronary heart disease (CHD), other clinical evidence of atherosclerosis, diabetes mellitus, or calculated 10-year CHD risk score >20%) following advice on a lipid-lowering diet with lipid-lowering treatment or if they had elevated plasma triglyceride concentrations (≥1.7 mmol/L) following advice on a lipid-lowering diet with or without lipid-lowering treatment.

#### 2.1.2. Exclusion Criteria

Exclusion criteria included a history of myocardial infarction, stroke, coronary artery bypass surgery or other revascularization procedures, unstable angina, or angioplasty within 3 months of screening; elevated liver enzymes (alanine aminotransferase [ALT] > 1.5 × ULN) or renal impairment (plasma creatinine > 200 *μ*mol/L) or uncontrolled endocrine or metabolic disease known to influence serum lipids or lipoproteins; a history of alcohol or drug abuse; a history of hypersensitivity to any of the ingredients contained in the study herb formula; women who were pregnant or lactating; initiation of lipid-lowering therapy or antidiabetic treatment within 4 weeks prior to screening. Subjects were excluded if they took weight lowering agents with 6 months prior to screening or were currently engaging in vigorous exercise or aggressive diet regimens for weight control.

The study protocol and statement of informed consent were approved by the Joint Clinical Research Ethics Committee of The Chinese University of Hong Kong and New Territories East Cluster before the start of the study. The study was conducted in compliance with the Declaration of Helsinki and all participants gave written informed consent.

### 2.2. Study Medication

The herbal formula (blood fat droplets (control)) and matching placebo were manufactured and supplied by Vita Green Pharmaceutical (HK) Ltd (Hong Kong, China). This product is registered for use as a natural health product in Hong Kong. Pretreated* Crataegus pinnatifida*,* Alisma orientalis*,* Polygonum multiflorum*,* Ganoderma lucidum,* and* Stigma maydis* were extracted with water:  ethanol (1 : 1) at 60°C and then extracted again with water alone. The extract was then concentrated.* Morus alba* was extracted with water at 80°C and then concentrated. The extracts of* Crataegus pinnatifida*,* Alisma orientalis*,* Stigma maydis*,* Polygonum multiflorum*,* Ganoderma lucidum*, and* Morus alba* were mixed (in a ratio of 3 : 2 : 2 : 1 : 1 : 1), vacuum dried, and ground into powder. The dosages of each herb in the final herb formula are shown in Table 1.

Ursolic acid was used as the quality marker of this herbal product and was quantified by HPLC. The mobile phase contained methanol-water-glacial acetic acid (88 : 12 : 0.2). UV detection was performed at 215 nm. The sample was defatted with petroleum ether and extracted using diethyl ether. The solvent was evaporated and the residue dissolved in methanol for injection. The specification is >0.01% ursolic acid in the final product. Heavy metal and toxic elements including arsenic, lead, and mercury were measured using in house methods. Microbial examination (total aerobic count, moulds and yeast count, and* Escherichia coli*) and pesticides residue analysis were also performed on the samples.

The placebo capsule contained starch and artificial food colouring. The herb formula and placebo were identical in packing, appearance, and colour.

### 2.3. Study Protocol

This study was a prospective, randomized, double-blinded, placebo-controlled, and parallel design study. After completion of a 2-week placebo run-in period, eligible patients were randomly assigned to receive the herbal formula or placebo which was consumed twice daily with or without food, four capsules in the morning and four capsules in the evening for a period of 12 weeks.

Randomization was performed using Random Allocation Software (Version 1.0, Isfahan University of Medical Sciences, Isfahan, Iran) that allows random lists to be generated with permuted block and designated seeds. Electronic and paper records of the randomization seed number and the randomization sequence were kept in the study center for operation. The allocation sequence was generated by Miao Hu (the first author), whereas participant enrollment and assignment were conducted by Brian Tomlinson and the study nurses. The patients, investigators, and the study staff were blinded to treatment assignment until the outcome assessment was completed.

Patients were assessed at baseline and at 6 and 12 weeks after the initiation of treatment with herb formula or placebo, with the last dose being consumed the evening before the visits. Anthropometric measurements, including body weight, waist circumference, hip circumference, and estimation of percentage body fat using an impedance device (TANITA Body Composition Analyzer BF-350, Tokyo, Japan), were performed by a research nurse. Blood pressure was measured by a semiautomatic sphygmomanometer (Critikon Dinamap; GE Medical Systems Information Technologies, Louisville, KY, USA). Fasting blood samples were taken for lipid profile, fasting glucose, and laboratory safety tests at the study visit. Adherence to study medication and tolerability were assessed at study visits. All subjects were asked to maintain their usual diet and other aspects of lifestyle during the study.

### 2.4. Sample Size Estimation

Some previous randomized, placebo-controlled studies with* Crataegus pinnatifida* or other herbs showed that a minimal number of 10–30 patients in each group were needed to demonstrate the significant effect of herb supplements on lipid and glucose levels depending on the effect of the herbs [30, 31, 36], but clinical data on the lipid-lowering effect of this herb product or with other similar herbal formulas in patients with dyslipidemia is still lacking. Assuming that this herb formula would show a mean of 20% greater reduction in LDL-C than placebo with the SD of the % reduction in LDL-C being 4% as some animal studies showed that some of these herbs might decrease plasma lipids by up to over 30% [35], 16 subjects in each group would be needed for 80% power with *α* = 0.05. Considering a drop-out rate of 20%, we planned to recruit at least 40 patients with dyslipidemia in the present study.

### 2.5. Biochemistry Measurements

All biochemistry tests including lipid profiles, glucose, glycated haemoglobin (HbA1c), and laboratory safety parameters (e.g., creatinine, creatine kinase, total bilirubin, alkaline phosphatase (ALP), and ALT) were performed by standard methods in the Chemical Pathology laboratory at the Prince of Wales Hospital, which has international laboratory accreditation. Total cholesterol level was measured by the enzymatic method (Centrichem Chemistry System, Baker Instruments Co. Allentown). High-density lipoprotein cholesterol (HDL-C) level was determined by using the fractional precipitation of dextran sulphate with manganous ion. Triglyceride levels were measured by the glyceryl dehydrogenase reaction following the hydrolysis of the triglyceride (Centrichem Chemistry System, Baker Instruments Co., Allentown). LDL-C concentrations were calculated according to the Friedewald formula or directly measured if the triglyceride level was greater than 4.5 mmol/L [37].

### 2.6. Statistical Analysis

Per protocol analysis was performed in a blind manner in 40 patients who had completed all study visits. The primary end point of the study was percentage change in LDL-C from baseline at 12 weeks. The secondary end points included percentage changes in other lipid parameters, HbA1c, fasting plasma glucose, and laboratory safety tests at 6 and 12 weeks. Continuous variables were expressed as mean ± SD unless otherwise indicated. Skewed data were logarithmically transformed before analysis. The baseline characteristics and the primary and secondary outcomes between the two treatment groups were compared using Student's *t*-test for normally distributed parameters or Mann-Whitney test for continuous variables that could not be successfully transformed into normally distributed data and chi-square tests for categorical variables. Paired-sample *t*-test was performed to assess changes of parameters within the placebo or active treatment group. Differences were considered to be statistically significant, if the two-sided *P* value is <0.05. Statistical analysis was performed using the Statistical Package for the Social Sciences version 17.0 (SPSS Inc., Chicago, IL, USA).

## 3. Results

### 3.1. Characteristics of Study Participants

**Figure 2:**
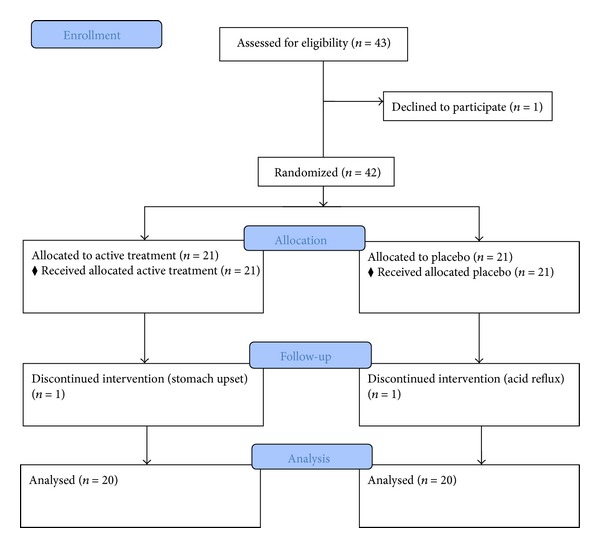
CONSORT flowchart of study recruitment and completion of the study.

A total of 43 patients were recruited for the study and 1 patient withdrew consent. A total of 42 patients were randomized and two patients dropped out due to adverse effects (one in each group) (Figure 2). Forty patients completed all study-related visits. Of those patients who completed the study, 16 (40%) were male, 19 (47.5%) had type 2 diabetes, and 12 (30%) had familial hypercholesterolemia (Table 2). The mean (±SD) age was 56 ± 8.8 years and the body mass index was 26.5 ± 3.9 kg/m^2^. Seventeen patients were on a background of stable lipid-lowering treatment with 14 of them receiving statins and 3 patients receiving gemfibrozil. There were no statistically significant differences in the baseline characteristics between the two groups except that the baseline LDL-C levels were higher in the active treatment groups than those in the placebo group (3.72 ± 1.17 versus 3.04 ± 0.82 mmol/L, *P* < 0.05).

### 3.2. Effects on Lipids and Glucose Levels

**Figure 3:**
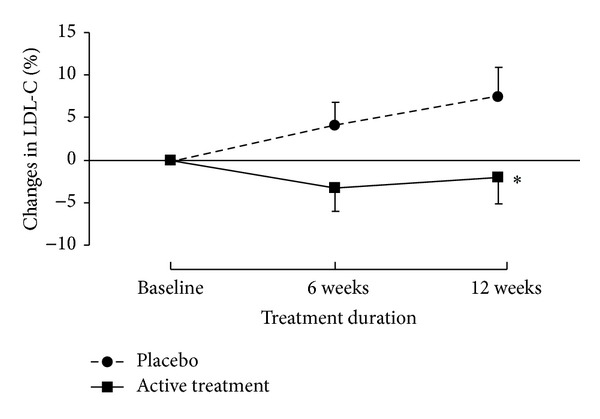
The percentage changes from baseline in LDL-C after 6 and 12 weeks of treatment. *P < 0.05.

There were no significant changes in body weight between baseline and after treatment for each of the groups during the study (Table 3). The baseline LDL-C levels were not associated with the LDL-C response to the active treatment and placebo (data not shown). After 6 and 12 weeks of treatment, the LDL-C levels increased by 4% and 7.4% in the placebo group, while they decreased by −3% and −2% in the active treatment group, and the difference in changes in LDL-C at week 12 between placebo and active treatment groups was significant (*P* = 0.062 and *P* < 0.05 versus placebo, after 6 and 12 weeks, resp.) (Figure 3). The mean level of triglycerides tended to be decreased in the placebo group and increased in the active treatment after 12 weeks of therapy, but this change was not statistically different compared to baseline or compared to the alternative regimen. The increase in the active treatment group was largely driven by an outlier who had an increase of triglycerides from 2.3 mmol/L to 8.5 mmol/L at the end of the study. His triglycerides after 6-week treatment with the herb product was 2.0 mmol/L suggesting that the increase in triglycerides at the end of study is likely related to changes in diet or other aspects of lifestyle which occurred during the longer follow-up period.

**Figure 4:**
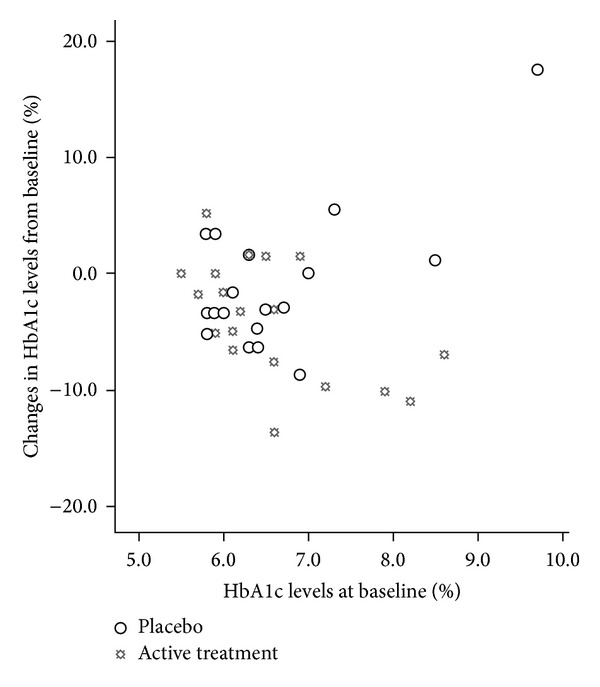
Association between the baseline levels of glycated haemoglobin and the percentage changes in glycated haemoglobin after 12 weeks of treatment.

There was no significant difference in the changes in other lipid parameters and fasting plasma glucose between the two groups (Table 3). The HbA1c levels significantly decreased by −3.9% in the active treatment group after 12 weeks of treatment, but this was not significantly different from the change with placebo (−1.1%) (*P* = 0.098). There was an inverse correlation between baseline HbAlc levels and the changes in HbAlc in the active treatment group (*r* = −0.565, *P* = 0.01), so that patients with higher baseline levels tended to show a greater fall, but this was not seen in the placebo group with or without including one outlier (*P* > 0.05) (Figure 4). Background lipid-lowering treatment or antidiabetic treatment had no effect on the lipid and glucose response to the treatment (data not shown).

### 3.3. Adverse Effects

Both active treatment and placebo were well tolerated. Plasma biochemical parameters in the two groups are shown in Table 4. All the laboratory safety parameters were within the normal range during the study. The plasma urea level in the placebo group was increased by 10.2%, which was significantly different from the decrease of −3.6% in the active treatment group (*P* < 0.05). There was a significant increase in the plasma albumin levels in the active treatment group but no difference in the changes in albumin levels between the two groups.

A total of 28 adverse events were reported by 23 participants (12 in the placebo group and 11 in the active treatment group). Of the two patients who withdrew from the study, one patient randomized to the active treatment complained of stomach upset and one patient randomized to placebo developed acid reflux. Influenza and cough were the most common adverse events (*n* = 8) followed by shoulder or knee pain (*n* = 5) and headache (*n* = 3). None of the adverse events were considered clinically significant. There was no statistically significant difference in rates of any adverse events among the treatment groups.

## 4. Discussion

In this randomized clinical study of a multiherb product containing* Crataegus pinnatifida*,* Alisma orientalis*,* Stigma maydis,* and other herbs which have long been considered to have hypolipidaemic and/or hypoglycaemic effects, there was a significant improvement in plasma LDL-C levels in patients receiving the active treatment for 12 weeks compared to those assigned to placebo (difference 9.4%) and this trend was already observed at 6 weeks. However, the change in LDL-C in the active treatment group is very small and the difference in the LDL-C response in the two groups may be largely driven by the increase in the LDL-C levels in the placebo group and may be influenced by the difference in baseline LDL-C levels.

It was shown that hawthorn fruit drink 250 mL (containing 1.4 mg hawthorn flavones) twice daily significantly decreased the plasma LDL-C, apolipoprotein B, and triglycerides by 10.4%, 7.4%, and 9.3%, respectively, in 30 Chinese patients with dyslipidemia in an early uncontrolled study [22], although another placebo-controlled study showed a Chinese therapeutic food supplement with hawthorn fruit and Chinese kiwifruit-extract compound had no effect on plasma LDL-C or triglyceride levels but it increased the HDL-C by 5% in Caucasian patients with dyslipidemia [38]. Several factors may contribute to the limited lipid-lowering effect of the tested herbal product in the present study. Insufficient dosing is one of the possibilities. Furthermore, the relatively mild elevated baseline plasma levels of LDL-C and background of lipid-lowering drugs may also influence the lipid-lowering efficacy although there was no significant association between the baseline LDL-C level, the baseline lipid-lowering treatment, and the lipid responses to the herb product. Another potential confounder is that our study recruited a rather heterogeneous group of patients receiving different treatments for their comorbidities, and this may contribute to the variability of the lipid response to treatment and limit the power of the study to detect a significant effect of the supplement. However, herbal supplements are often used concomitantly with conventional drugs, especially in the elderly or those with chronic disease such as dyslipidemia and diabetes, and the goal of the study was to evaluate the real-world effect of the herbal product.

There was no statistically significant difference in the percentage change in HbA1c levels between the herb formula and placebo during the study, although there was a significant decrease in HbA1c levels in the active treatment group, particularly in patients with higher baseline levels, which is a typical finding with many antihyperglycaemic drugs. This result may suggest a potential beneficial effect of this supplement on the overall glycaemic control in patients with abnormal metabolic states of glucose regulation, for example, impaired glucose tolerance and diabetes. Several lines of evidence suggest the effectiveness of* Crataegus pinnatifida*,* Alisma orientalis*, and other herbs contained in this herb supplement on glucose and lipid metabolism [5, 6, 12, 24]. Flavonoids and triterpenes appear to be the main active components of these herbs to exert antihyperlipidaemia and antihyperglycaemia effect. However, this study was not designed to examine the effect of this herb formula in treating diabetes. Further research with larger sample size is needed to investigate the hypoglycaemic effect of this supplement in patients with diabetes.

This study has several limitations which need to be considered. Firstly, there was significant difference in the baseline LDL-C levels between the placebo group and the active treatment group. This may be related to the small sample size. In this study, patients were selected if they had either elevated LDL-C and/or elevated triglycerides, and thus they may have different types of dyslipidemia, contributing to the different lipid profiles of the two groups in this small study. The small sample size is another major limitation of the study. The studied herb formula only showed a marginal effect on plasma LDL-C levels compared to placebo and the study is underpowered to detect small differences between the two groups. In addition, it would be useful to measure various apolipoprotein levels, for example, apoAI, apoB, and apoE, although these apolipoprotein levels are usually closely associated with particular plasma lipid levels. It would be helpful to identify and quantify the active components in this herbal formula and to test whether these active components reduce the plasma lipids in a dose-dependent manner, but the study was designed to examine the real-world lipid-lowering effect of this commercially available herbal product in patients with dyslipidemia using a recommended dose, and thus the dose-response was not assessed.

In conclusion, this randomized, placebo-controlled study conducted in an ambulatory outpatient setting showed a marginal beneficial effect of the multiherb formula on reducing plasma LDL-C levels in subjects with dyslipidemia without any noticeable adverse effects. The finding was consistent with some of the previous experimental* in vitro* and animal studies with these herbs. Further well-designed clinical studies are warranted to support or refute the clinical use of herbal medicines in reducing cardiovascular risk.

## Figures and Tables

**Table 1:** The multiherb formula tested in the study.

Herb extracts (Chinese pinyin names)	Weight (mg per capsule)	Proportions
*Crataegus pinnatifida* (Shan Zha)	129	30%
*Alisma orientalis* (Ze Xie)	86	20%
*Stigma maydis* (Yu Mi Xu)	86	20%
*Ganoderma lucidum* (Ling Zhi)	43	10%
*Polygonum multiflorum* (He Shou Wu)	43	10%
*Marus alba* (Sang Ye)	43	10%

**Table 2:** Baseline characteristics of the study participants.

	Placebo (*n* = 20)	Herb formula (*n* = 20)
Age, years	54.7 ± 9.1	57.5 ± 8.4
Males, *n* (%)	10 (50)	6 (30)
Body weight, kg	71.3 ± 17.2	65.0 ± 10.7
Body mass index, kg/m^2^	27.2 ± 4.7	25.9 ± 2.8
Body fat, %	31.5 ± 7.3	31.9 ± 6.1
Waist, cm	94.3 ± 12.7	87.7 ± 7.7
SBP, mmHg	119.5 ± 12.4	118.9 ± 10.8
DBP, mmHg	77.1 ± 9.6	73.6 ± 9.6
Pulse, bpm	71.1 ± 10.1	67.4 ± 7.5
Diabetes, *n* (%)	8 (40)	11 (55)
Hypertension, *n* (%)	14 (70)	10 (50)
FH, *n* (%)	7 (35)	5 (25)
On lipid-lowering treatment	9 (45)	8 (40)
On antidiabetic treatment	5 (25)	5 (25)
Baseline TC, mmol/L	5.39 ± 0.89	5.90 ± 1.17
Baseline HDL-C, mmol/L	1.29 ± 0.39	1.28 ± 0.25
Baseline TG, mmol/L	2.35 ± 1.18	2.00 ± 0.71
Baseline LDL-C, mmol/L	3.04 ± 0.82	3.72 ± 1.17*
Baseline non-HDL-C, mmol/L	4.10 ± 0.68	4.63 ± 1.13
Fasting glucose, mmol/L	5.57 ± 1.23	5.84 ± 2.17
HbA1c, %	6.57 ± 0.98	6.59 ± 0.83

DBP: diastolic blood pressure; TC: total cholesterol; FH: familial hypercholesterolaemia; HDL-C: high-density lipoprotein cholesterol; LDL-C: low-density lipoprotein cholesterol; SBP: systolic blood pressure; TG: triglycerides.

*P < 0.05 versus placebo.

**Table 3:** Changes in body weight, lipids, and glucose at week 12.

	Placebo (*n* = 20)			Herb formula (*n* = 20)			*P* value (versus placebo)
	Baseline	Week 12	% change	Baseline	Week 12	% change	
Body weight, kg	71.3 ± 17.2	69.0 ± 20.9	−2.7 ± 15.0	65.0 ± 10.7	65.7 ± 10.0	0.8 ± 1.9	0.310
TC, mmol/L	5.39 ± 0.89	5.61 ± 1.01	4.5 ± 10.3	5.90 ± 1.17	5.96 ± 1.21	1.4 ± 10.5	0.362
HDL-C, mmol/L	1.29 ± 0.39	1.34 ± 0.46	0.7 ± 12.4	1.28 ± 0.25	1.27 ± 0.30	2.0 ± 12.4	0.276
TG, mmol/L	2.35 ± 1.18	2.21 ± 1.04	−6.7 (−18.3, 15.8)	2.00 ± 0.71	2.42 ± 1.61	5.8 (−14.8, 16.3)	0.383
LDL-C, mmol/L	3.04 ± 0.82	3.27 ± 0.95*	7.4 ± 15.5	3.72 ± 1.17	3.64 ± 1.17	−2.0 ± 13.6	0.049
Non-HDL-C, mmol/L	4.10 ± 0.68	4.28 ± 0.78	3.6 ± 9.2	4.63 ± 1.13	4.69 ± 1.16	0.4 ± 12.5	0.368
Fasting glucose, mmol/L	5.57 ± 1.23	5.80 ± 2.02	2.9 ± 12.1	5.84 ± 2.17	5.80 ± 1.19	0.3 ± 7.8	0.432
HbA1c, %	6.57 ± 0.98	6.54 ± 1.36	−1.1 ± 5.7	6.59 ± 0.83	6.31 ± 0.65**	−3.9 ± 4.9	0.098

TC: total cholesterol; HDL-C: high-density lipoprotein cholesterol; LDL-C: low-density lipoprotein cholesterol; TG: triglycerides.

Data are presented as mean ± SD or median (interquartile range).

*P < 0.05; **P < 0.01 compared to baseline.

**Table 4:** Changes in biochemical parameters at week 12.

	Placebo (*n* = 20)			Herb formula (*n* = 20)			*P* value (versus placebo)
	Baseline	Week 12	% change	Baseline	Week 12	% change	
Creatinine, *μ*mol/L	70.5 ± 13.1	68.1 ± 14.2	−3.3 ± 9.5	68.7 ± 12.5	66.7 ± 12.6	−2.5 ± 9.9	0.799
Creatine kinase, U/L	144.3 ± 72.8	135.7 ± 78.7	−1.0 (−26.8, 10.1)	144.3 ± 101.4	141.2 ± 73.6	1.2 (−7.5, 15.0)	0.563
Total protein, g/L	75.2 ± 3.3	76.1 ± 3.1	1.3 ± 3.7	74.4 ± 4.2	75.1 ± 3.3	1.1 ± 3.8	0.860
Urea, mmol/L	4.78 ± 1.18	5.13 ± 1.15	10.2 ± 24.6	5.35 ± 1.29	5.13 ± 1.36	−3.6 ± 15.6	0.041
Albumin, g/L	44.9 ± 2.4	45.3 ± 2.2	0.9 ± 4.5	43.5 ± 2.7	45.0 ± 2.6**	3.6 ± 5.4	0.092
Bilirubin, *μ*mol/L	12.6 ± 5.5	13.1 ± 6.8	4.9 ± 25.2	12.1 ± 4.0	11.9 ± 5.2	1.4 ± 32.2	0.706
ALT, IU/L	31.4 ± 9.9	32.1 ± 14.3	3.2 ± 26.1	24.7 ± 9.8	26.6 ± 10.0	17.7 ± 60.5	0.332
ALP, U/L	73.8 ± 15.8	73.9 ± 14.2	0.8 ± 7.5	59.5 ± 16.8	60.0 ± 14.4	2.1 ± 11.5	0.672
Urate, mmol/L	0.38 ± 0.09	0.38 ± 0.08	3.1 ± 12.6	0.34 ± 0.06	0.35 ± 0.07	0.8 ± 13.2	0.567

ALT: alanine aminotransferase; ALP: alkaline phosphatase; data are presented as mean ± SD or median (interquartile range); **P < 0.01 compared to baseline.
